# Increasingly expanded future risk of dengue fever in the Pearl River Delta, China

**DOI:** 10.1371/journal.pntd.0009745

**Published:** 2021-09-24

**Authors:** Wei Wu, Hongyan Ren, Liang Lu

**Affiliations:** 1 State Key Laboratory of Resources and Environmental Information System, Institute of Geographic Sciences and Natural Resources Research, Chinese Academy of Sciences, Beijing, China; 2 School of Geography and Ocean Science, Nanjing University, Nanjing, China; 3 Key Laboratory of Coastal zone Development and Protection, Ministry of Land and Resources of China, Nanjing, China; 4 State Key Laboratory of Infectious Disease Prevention and Control, National Institute for Communicable Disease Control and Prevention, Chinese Center for Disease Control and Prevention, Beijing, China; University of California, Davis, UNITED STATES

## Abstract

**Background:**

In recent years, frequent outbreaks of dengue fever (DF) have become an increasingly serious public health issue in China, especially in the Pearl River Delta (PRD) with fast socioeconomic developments. Previous studies mainly focused on the historic DF epidemics, their influencing factors, and the prediction of DF risks. However, the future risks of this disease under both different socioeconomic development and representative concentration pathways (RCPs) scenarios remain little understood.

**Methodology and principal findings:**

In this study, a spatial dataset of gross domestic product (GDP), population density, and land use and land coverage (LULC) in 2050 and 2070 was obtained by simulation based on the different shared socioeconomic pathways (SSPs), and the future climatic data derived from the RCP scenarios were integrated into the Maxent models for predicting the future DF risk in the PRD region. Among all the variables included in this study, socioeconomics factors made the dominant contribution (83% or so) during simulating the current spatial distribution of the DF epidemics in the PRD region. Moreover, the spatial distribution of future DF risk identified by the climatic and socioeconomic (C&S) variables models was more detailed than that of the climatic variables models. Along with global warming and socioeconomic development, the zones with DF high and moderate risk will continue to increase, and the population at high and moderate risk will reach a maximum of 48.47 million (i.e., 63.78% of the whole PRD) under the RCP 4.5/SSP2 in 2070.

**Conclusions:**

The increasing DF risk may be an inevitable public health threat in the PRD region with rapid socioeconomic developments and global warming in the future. Our results suggest that curbs in emissions and more sustainable socioeconomic growth targets offer hope for limiting the future impact of dengue, and effective prevention and control need to continue to be strengthened at the junction of Guangzhou-Foshan, north-central Zhongshan city, and central-western Dongguan city. Our study provides useful clues for relevant hygienic authorities making targeted adapting strategies for this disease.

## Author summary

As the most prevalent mosquito-borne infection in the world, the dengue fever (DF) outbreaks were increasingly serious in China in recent years. In particular, almost 85% of the total cases in the mainland China were reported in the PRD region, which has been attributed in previous studies to its suitable subtropical climate with hot, wet summers and mild autumns, as well as the highest urbanization, crowded and large floating population in some informal urban settlements. In consideration of an expected rapid socioeconomic development in the PRD region in the future, the climatic and socioeconomic variables were comprehensively integrated into the ecological niche models (ENM) for projecting the spatial distribution of DF risk in 2050 and 2070. The results showed that more detailed geographic distribution of DF risk zones (grids) was appropriately identified for the current and future scenarios through the models aggregating both climatic and socioeconomic variables. In comparison, the socioeconomic variables presented better capability of identifying the risk zones in detail. Along with continuously rapid socioeconomic development and global warming in the future, the PRD region may be confronted by increasing zones and population of high/moderate DF risk, as well as their spatial expansion during the 2050 and 2070. Accordingly, a forward-looking public health planning should be appropriately made so as to address the DF risk posed by global warming and the expected socioeconomic growth in this region in the future.

## Introduction

Dengue fever (DF) is an infectious disease caused by four serotypes (Dengue virus [DENV] 1–4), which are principally transmitted by *Aedes aegypti* and *Aedes albopictus* mosquitoes [1]. Dengue is endemic in more than 100 countries in tropical and subtropical areas around the world, especially in Southeast Asia, the Americas, the Western Pacific, Africa, and Eastern Mediterranean regions [1]. In the past 50 years, the incidence of dengue has increased 30-fold with human population growth, increased urbanization, expanded distributions of the mosquito vectors, and population movement [2]. It is estimated that about 5–6 billion people (50–60% of the projected global population) will be at risk for dengue transmission by 2085 [3].

There were no documented cases of DF in China until an outbreak in Foshan, Guangdong Province occurred in 1978. Subsequently, DF epidemics have occurred once every 4–7 years, which has gradually attracted widespread attention throughout the mainland China. From 2003 to 2014, more than 55,000 cases were reported in mainland China. Approximately 94% of the local cases in this period occurred in Guangdong Province, and most of these cases (> 90%) were located in the Pearl River Delta (PRD). The 2014 dengue outbreak in Guangzhou Province was particularly severe [4–7].

Previous studies have indicated that dengue remains an imported disease in China. The temporal and spatial heterogeneity of DF incidence is related to risk factors, such as local climate, geography, environment, and socioeconomic status of the affected human population [8–11]. Local climatic and environmental conditions, such as precipitation, temperature, and humidity have been shown to determine the size of DF outbreaks [8,9]. DF outbreaks may also be related to socioeconomics factors such as population size, affluence, and access to public transportation [10,11]. These studies documented environmental and socioeconomic factors associated with DF in the PRD, and stressed the need to understand the spatial and temporal impacts of risk factors on the geographical distribution of epidemics [10,11].

There are no effective vaccines or specific therapies available to stop the rapid worldwide spread of DF [12]. Therefore, knowledge of the relevant factors influencing DF epidemics, in a temporal and spatial context, may allow better prediction of outbreaks and more effective prevention and control measure. Studies have used the time series Autoregressive Integrated Moving Average (ARIMA) model [13,14], the back propagation (BP) neural network model [15], the generalized additive model (GAM) [16,17], and the species ecological niche model [18,19] to predict the spatial and temporal distribution of dengue epidemics. For example, Gharbi M. et al. [13] proposed that Seasonal ARIMA models, using climatic data as independent variables, could be incorporated into a reliable monitoring system of dengue outbreaks. Ren et al. [15] used population density, land use data, and climatic data to build a BP neural model and predict the spatial distribution of DF in the Guangzhou-Foshan region. Zheng et al. [16] used a GAM to identify potential climatic and socioeconomic factors that influence spatiotemporal DF patterns in typical DF epidemic regions of China. Machado-Machado et al. [19] demonstrated that climatic variables were more important determinants of suitability for DF in Mexico than the socioeconomic variables based on the species distribution model. These estimates have yielded similar results related to the extent of dengue transmission but there is less agreement regarding specific geographic patterns or the potential for range contraction.

Within the context of continued global warming and urbanization, it is valuable to evaluate if the DF risk area in the PRD region will continue to expand and to determine the major factors influencing factors its spread. We reviewed previously published methods that successfully mapped the distribution of dengue [11]. We used the maximum entropy (Maxent) ecological niche model, combined with DF cases and climatic and socioeconomic environmental data, to analyze and predict the risk area of dengue in current and future scenarios within the PRD region, which will provide references and support for local monitoring and prevention of future dengue epidemics.

## Materials and methods

### Study area

**Fig 1:**
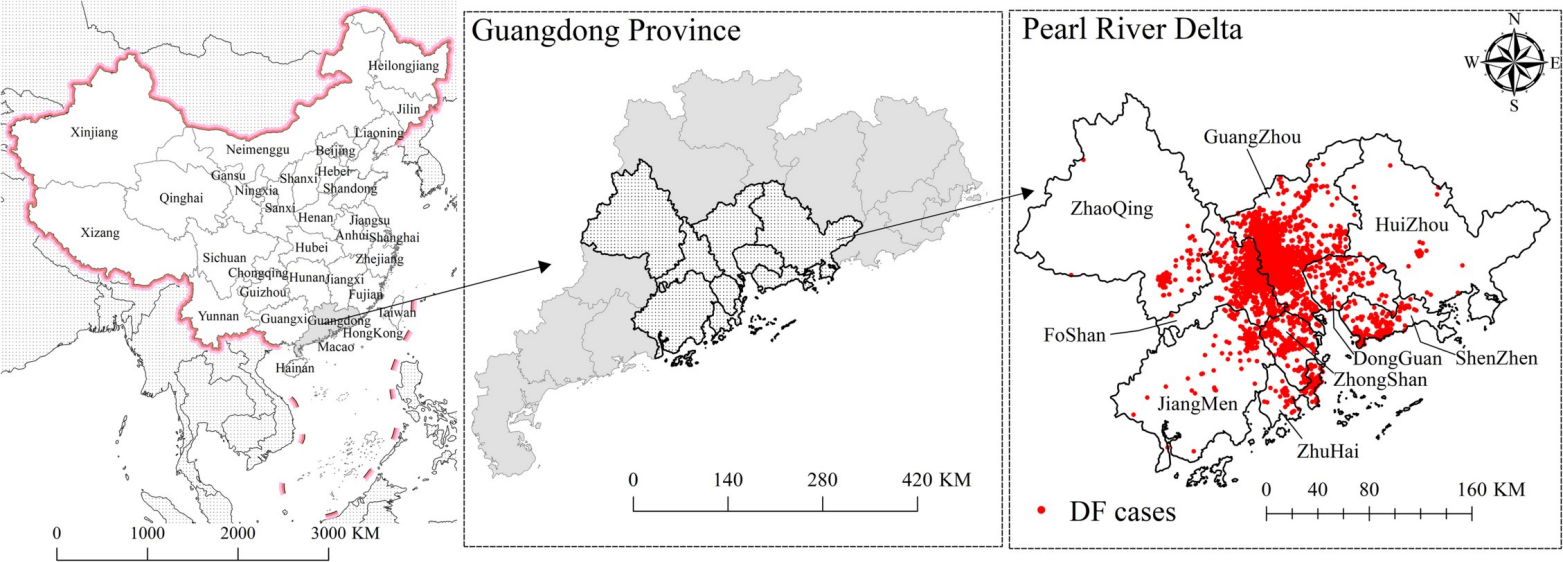
Study areas and the dengue cases of 2014 distributed in the PRD. https://www.resdc.cn/data.aspx?DATAID=201.

The PRD is the low-lying area surrounding the Pearl River estuary, located in southern China, adjacent to Hong Kong and the Macao Special Administrative Region (Fig 1). It has an area of 44,700 square kilometers with a population of 58.74 million (as of the end of 2015). This highly developed region contains nine densely populated urban centers, including Guangzhou, Foshan, and Shenzhen [20]. As one of the main transport hubs between Mainland China and abroad, the PRD has strong population mobility and the development plan of Guangdong-Hong Kong-Macau Greater Bay Area has risen to the national strategy [21]. Meanwhile, the PRD has a humid subtropical climate with hot, wet summers and mild, dry winters. These suitable natural and social environmental conditions are favorable to the growth of *Ae*. *albopictus*, which is the sole vector of DENV in the PRD, and to the transmission of DENV, making it a high-risk area for DF [22].

### Dengue data collection and processing

DF is a notifiable disease in China, which means that, once diagnosed, cases must be reported to the web-based National Notifiable Infectious Disease Reporting Information System (NIDRIS) within 24 h [7]. The DF case information includes age, sex, address, and time of onset. The DF epidemic data for this study were obtained from the China Center for Disease Control and Prevention, covering 47,466 DF case data from 2003–2014 (S1 Fig). The targeted DF cases in our study included clinically diagnosed (based on clinical manifestations and epidemiologic exposure history) or laboratory-confirmed cases (“clinically diagnosed cases presenting with any of the following lab test results relating to DF: a 4-fold increase in specific IgG antibody titer, positive on a PCR test or viral isolation and identification test”). The address information of the confirmed cases, after deidentification, was used in conjunction with geocoding and coordinate deviation correction to produce case data for a spatial point layer using ArcGIS 10.6 (ESRI, Redlands, CA, USA) software [23]. In 2014, DF cases in the Guangdong Province reached a peak, with a total of 44,270 cases reported, of which 42,963 were from the PRD area, accounting for 97.05% of the entire Guangdong province.

### Climate scenarios and variables

In the Fifth Assessment Report, the Intergovernmental Panel on Climate Change

(IPCC) combined different sets of potential economic, technological, demographic, policy, and institutional futures, leading to the distinction of four representative concentration pathways (RCPs) (i.e., RCP8.5, RCP6.0, RCP4.5, and RCP2.6) representing the specific cumulative estimate of radiative forcing (greenhouse gas emissions from all human sources) by the year 2100, expressed in watts per square meter (W m^−2^). RCP8.5 is characterized by increasing greenhouse gas emissions and representative of scenarios that lead to high greenhouse gas concentration levels [24]. RCP6.0 is considered a ‘stabilization scenario’ with an emissions peak around 2080 and then a decline due to the application of a range of technologies and mitigating strategies and stabilization shortly after 2100 [25]. RCP4.5 is also a stabilization scenario, resulting from aggressive greenhouse gas reduction measures and an emissions peak of around 2040 and stabilization shortly after 2100 [26]. RCP2.6 represents an aggressive mitigation scenario, with radiative forcing peaking at around 3.1 W m^−2^ by 2050, and returning to 2.6 W m^−2^ by 2100 [27].

In this study, we present possible dengue futures based on RCPs 2.6, 4.5, and 8.5 scenarios. The climatic data are a combination of temperature, rainfall, and humidity using multi-year average meteorological data (Table 1). This data yielded 19 bioclimatic variables with a spatial resolution of 30 arc seconds (i.e., 1km×1km per grid resolution). Data were obtained from WorldClim [28], including three time periods: current period (1970–2010), 2050 period (2041–2060), and 2070 period (2061–2080).

**Table 1:** Different types of data used in this study.

Description	Abbreviation /Type	Source
Annual Mean Temperature	bio1/Continuous	WorldClim
Mean Diurnal Range (Mean of monthly (max temp—min temp))	bio2/Continuous	
Isothermality (BIO2/BIO7) (* 100)	bio3/Continuous	
Temperature Seasonality (standard deviation *100)	bio4/Continuous	
Max Temperature of Warmest Month	bio5/Continuous	
Min Temperature of Coldest Month	bio6/Continuous	
Temperature Annual Range (BIO5-BIO6)	bio7/Continuous	
Mean Temperature of Wettest Quarter	bio8/Continuous	
Mean Temperature of Driest Quarter	bio9/Continuous	
Mean Temperature of Warmest Quarter	bio10/Continuous	
Mean Temperature of Coldest Quarter	bio11/Continuous	
Annual Precipitation	bio12/Continuous	
Precipitation of Wettest Month	bio13/Continuous	
Precipitation of Driest Month	bio14/Continuous	
Precipitation Seasonality (Coefficient of Variation)	bio15/Continuous	
Precipitation of Wettest Quarter	bio16/Continuous	
Precipitation of Driest Quarter	bio17/Continuous	
Precipitation of Warmest Quarter	bio18/Continuous	
Precipitation of Coldest Quarter	bio19/Continuous	
Population density	POP/Continuous	RESDC / GeoSOS
Road Density	ROAD/ Continuous	
Gross domestic product	GDP/ Continuous	
Land use and land cover	LULC/Categorical	
Digital Elevation Model	DEM/Continuous	
Slope	Slope/ Continuous	

### Socioeconomic scenarios and variables

#### Current socioeconomic variables

The population density, GDP, land use and land cover (LULC), digital elevation model (DEM) and slope data in 2010 were obtained from the Resources and Environmental Science Data Center (RESDC) [29]. Road density was generated from the road network vector data [30] including all roads in the PRD region, and was the ratio of the length of road in each grid to the area of the corresponding unit. The spatial resolution of the data for all variables was 1km×1km, and the detailed descriptions are shown in Table 1.

#### Future socioeconomic variables

The LULC data of 2050 and 2070 were obtained from the Geographical Simulation and Optimization System (GeoSOS) [31]. In addition, we attempted to predict GDP and population in the different Shared Socioeconomic Pathways (SSPs). Considering that the PRD region is already highly urbanized, this study assumed that the road network of PRD in the future will not change significantly.

The SSPs provide narrative descriptions and quantifications of possible developments of socioeconomic variables. The variables include population growth, economic development, and the rate of technology change and these characterize challenges to mitigation and to adaptation. These SSPs are based on results that may lead to more or less difficult climate change challenges. Therefore, these results can be combined with RCPs in a two-dimensional matrix to formulate multiple potential scenarios of global change to 2100 [32]. A previous study [33] suggested a set of suitable combinations of RCPs and SSPs that would allow comparison to the former scenarios presented in the 2000 IPCC Special Report on Emissions Scenarios (SRES). In this study, we chose the following combinations: (1) RCP8.5 with SSP3 to correspond with the SRES A2 world (increasing population and regionally-oriented economic development); (2) RCP4.5 with SSP2 to correspond with the SRES A1B/B2 world (both characterized by low population growth and technological change); (3) RCP2.6 with SSP1 to correspond with the SRES B1 world (global economic convergence and the introduction of resource-efficient technologies). The GDP and population data in the PRD from 2010 to 2100 under the different SSPs scenario (S2 Fig) were obtained from the Nanjing University of Information Science and Technology [34]. Using these projections, we carried out the following procedures in order to incorporate GDP per 1×1-km grid in our dengue models and projections.

#### GDP and population density per grid

The dominant factors affecting the spatial distribution of a population include climate, topography, hydrology, LULC, and traffic conditions. Among them, the land use type (especially cultivated land, residential sites and urban industrial and mining land) had the greatest impact on population distribution [35,36]. As such, we calculated the correlation between the different land use index (i.e., the ratio of different land use area to each county area) and the mean population density at the county level (Table A in S1 Text), and selected major land use indices that were more relevant to population density to construct a model between land use index and population density. We calculated the population density per 1×1-km grid based on the established model by linking the obtained future land use data and adjusted the total population for each scenario (SSPs 1–3) and year (2050 and 2070) according to the results of S2A Fig. Subsequently, we calculated the GDP per capita by dividing GDP by the total population for each scenario (SSPs 1–3) and year (2050 and 2070). We were then able to multiply GDP per capita by population density and produce the GDP per 1×1-km grid. Detailed information about the spatial distribution simulation of population density and GDP in the future can be found in S1 Text.

### Correlation analysis and comparative analysis of DF high risk

To explore the correlation between DF epidemics and different environmental variables, we used the Spearman correlation coefficient between the DF cases and all environmental variables in the 1×1-km grid based on SPSS 19.0 (SPSS Inc, Chicago, IL, USA) software. To analyze the trend of high-risk area of DF epidemic transmission, the high risk region of current and future time periods was extracted separately, and each of the two scenarios was divided into a group to further explore the spatial distribution of the risk differences between each group. This portion of the experiment was done based on the spatial analysis toolbox of ArcGIS 10.6 (ESRI, Redlands, CA, USA) software.

### Predicted modeling and validation

Ecological Niche Models (ENMs) are able to explore the non-random relationship between the disease and environmental factors based on known vectors, hosts, pathogens, and human case information. ENMs achieve fine-scale resolution of distributions limited only by the spatial precision of the input occurrence data and the input environmental variables datasets, and it can improve the spatial resolution in representing spatial patterns in disease risk [37]. Maxent is one of the most widely used ENM which is a general purpose machine-learning technique based on the principle of maximum entropy. Maxent offers several advantages that make it appropriate for this study: it is non-parametric, requires presence-only data, utilize both continuous and categorical data, incorporates interactions between variables, and produces continuous maps of suitability [38,39]. Maxent has been shown to perform as well as or better than other ENMs [40]. The maximum entropy estimation procedure assumes that the information to be modeled (i.e., DF case distribution) is incomplete and aims to incorporate the minimum amount of non-empirical information.

This study used the Maxent model to predict the spatial distribution of DF transmission spread risk based on all DF cases data from 2003~2013 (4,503) and 25% of DF cases data in 2014 (10,741), considering the DF outbreak in 2014 and avoiding the occurrence of overfitting. The remaining 75% of DF cases in 2014 (32,222) were used to validate the predicted risk areas by overlaying these DF case points onto the prediction maps. Empirical DF-case distribution was used to set a number of constraints on the maximum entropy distribution such that the expected value of each predictor variable under this estimated distribution equaled its mean in the empirical distribution. The maximum entropy distribution was estimated based on a maximum likelihood approach using a sequential-update algorithm that started from a uniform distribution and sequentially modified one or more weights of the predictor variables to maximize the average log probability of the presence samples. DF risk hierarchical maps divided into high (>0.5), moderate (0.35–0.5), low (0.05–0.35) and Zero (<0.05) levels [41,42].

In order to evaluate the model results, ten replicates of each model were generated by bootstrapping replicate modeling data where 75% of the DF case points were used for training and the remaining 25% were used for testing. All case points were merged with 10,000 randomly selected background points and were entered into a receiver operating characteristic (ROC) plot analysis to derive the area under the curve (AUC). AUC is a measure of performance that compares the model predictive ability [43]. Model accuracy using AUC was characterized as follows: 0.50–0.60, insufficient; 0.60–0.70, poor; 0.70–0.80, average; 0.80–0.90, good; and 0.90–1.00, excellent [44,45].

## Results

### Correlation analysis

**Fig 2:**
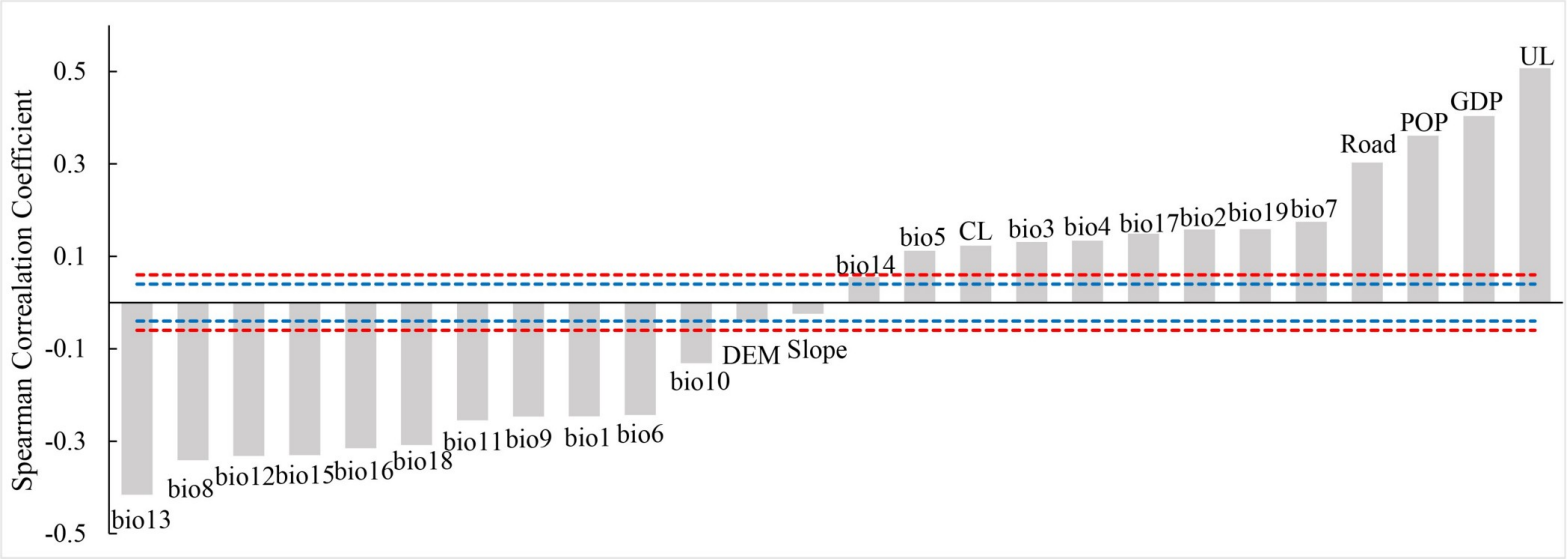
Correlation analysis between DF and different environmental variables. (Blue dotted line indicates p < 0.05 and the red dotted line indicates p < 0.01. CL: cultivated land, Road: road density, GDP: gross domestic product, POP: population density, UL: urban land).

The Spearman correlation coefficient (Fig 2) showed a significant correlation between all climatic variables and dengue cases. Most of the precipitation and temperature variables had a stronger negative correlation with the epidemic. The precipitation of the wettest month (i.e., bio13) (*r* = -0.42, P < 0.001) and mean temperature of wettest quarter (i.e., bio8) (*r* = -0.34, P < 0.001) contributed to the strongest negative correlation. In the socioeconomic environment variables, except for DEM and slope variables, the DF cases were strongly associated with the urban land (*r* = 0.51, P < 0.001), followed by GDP (*r* = 0.40, P < 0.001), population density (*r* = 0.36, P < 0.001), and road density (*r* = 0.30, P < 0.001), which were significantly greater than most climatic variables (except for bio13(*r* = -0.42), bio8 (*r* = -0.34), bio12 (*r* = -0.33), bio15 (*r* = -0.33), bio16 (*r* = -0.32), bio18 (*r* = -0.31)). According to the results of principal component analysis among different variables (S2 Text), it was difficult to determine which variable was excluded from the Maxent prediction model. Therefore, the input environmental variables in the subsequent DF epidemic prediction model included all climatic variables, LULC, GDP, road density, and population density.

### Socioeconomic development in the future

**Fig 3:**
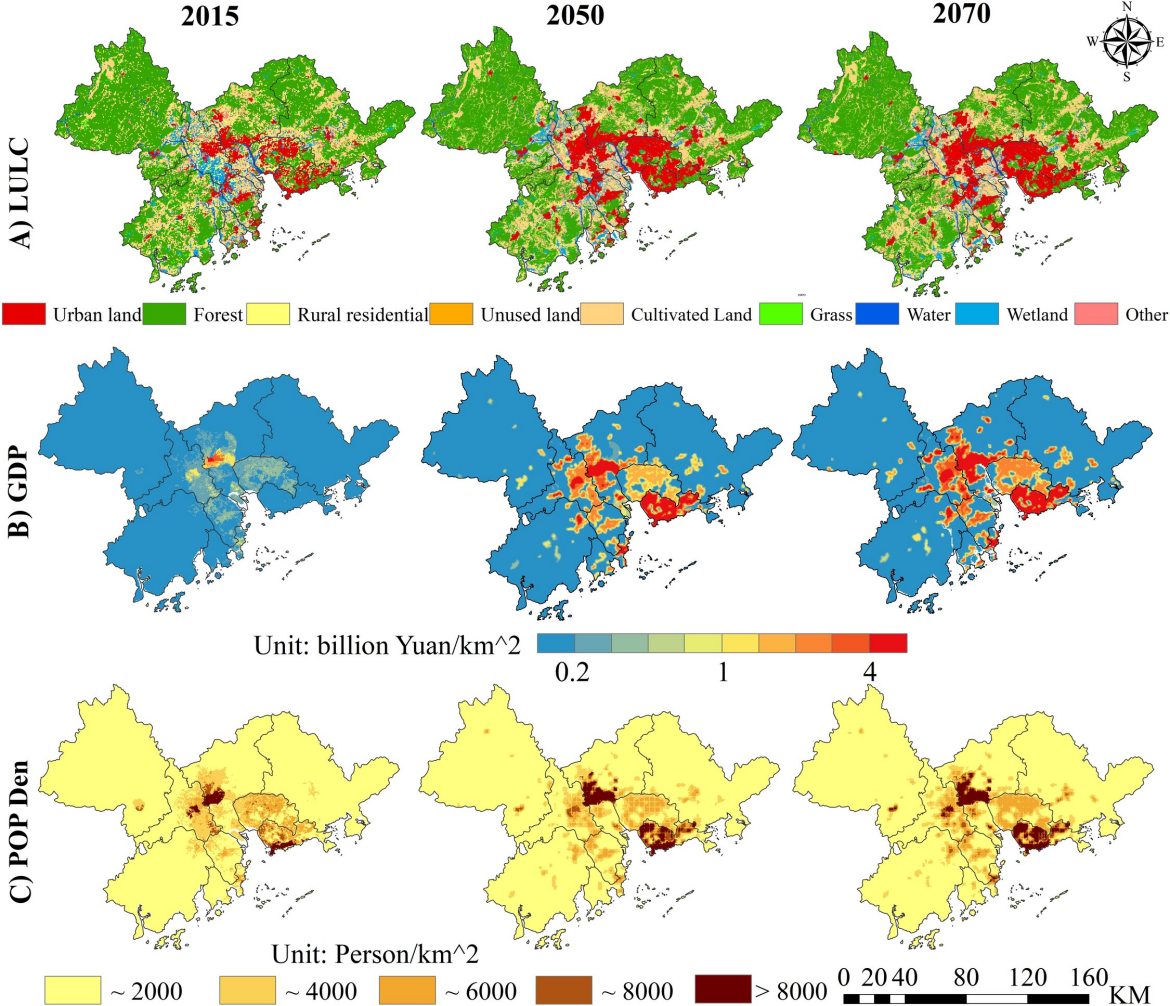
The spatial distribution of different socioeconomic variables in 2015, 2050 and 2070. (a) LULC, (b) GDP, and (c) population density. https://www.resdc.cn/data.aspx?DATAID=201.

According to the spatial-temporal distribution map of land use types (Fig 3A and S1 Table), the urban land will continue to expand from 2015 to 2050, while the rural residential, other construction land, cultivated land, grassland, water bodies, and wetland will shrink slightly. The rate of urban land expansion will slow down in 2070, and will continue to expand toward the southeast parts of Shenzhen and Dongguan. These results showed that, from 2015 to 2070, the land use and cover change in the PRD will be dominated by changes in urban land, and the high level of urbanization will gradually stabilize.

In this study, the GDP of the PRD region reached 15.27 trillion yuan in 2050 and 21.01 trillion yuan in 2070 (under the SSP2). Fig 3B shows that, during 2015–2070, the region of GDP growth included Shenzhen and counties at the junction of Guangzhou and Foshan. The total population of the PRD in 2015 was 51.87 million. The predicted total population under the SSP2 will be 74.84 million in 2050, but it will decrease to 76.03 million in 2070. The increased population will be mainly concentrated in the coastal areas of Shenzhen, the Baiyun District, and the Huangpu District of Guangzhou (Fig 3C).

### Model performance

The AUC values of three models were greater than 0.84, indicating that the prediction models fit well in the current scenarios (Table 2). When the socioeconomic variables were added to the climatic model, the AUC values of the predicted model increased slightly.

**Table 2:** Model validation results of the current situation.

Model	Mean AUC		DF cases in given risk area (% of Cases/ % area of the PRD)			
	Training AUC	Test AUC	Zero	Low	Moderate	High
A1	0.852	0.849	0.12/48.99	3.33/36.11	6.04/7.46	90.51/7.44
A2	0.854	0.852	0.40/60.12	3.32/26.80	5.88/5.09	90.40/8.00
A3	0.865	0.864	0.41/61.87	4.11/27.28	4.75/5.53	90.73/5.32

A1 is the climatic model, A2 is the socioeconomic model, A3 is the climatic and socioeconomic model.

**Fig 4:**
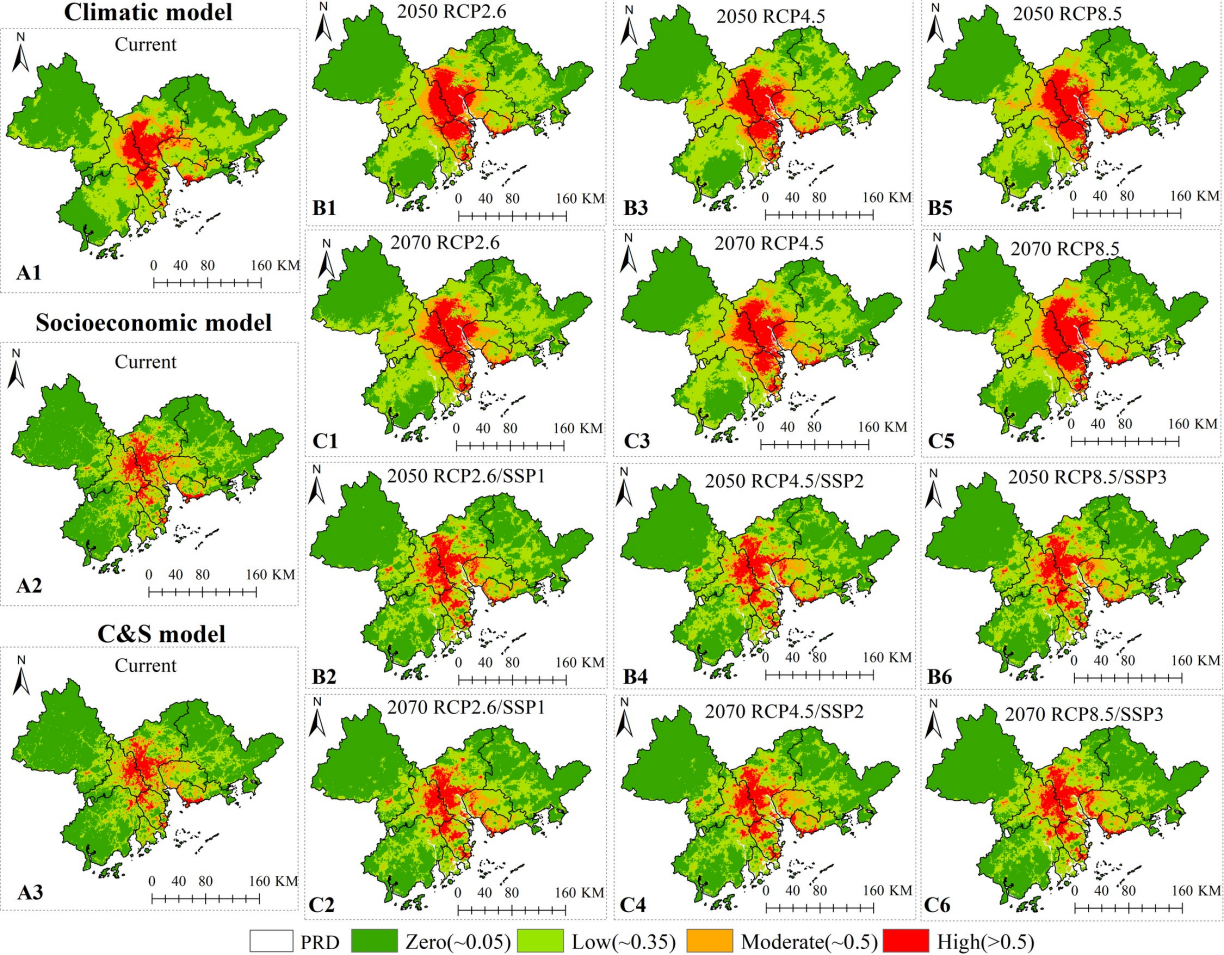
Predictive maps of current and future DF risk distribution in the different variables model. A1, B1, B3, B5, C1, C3, and C5 are the DF risk spatial distribution predicted by the climatic model. A3, B2, B4, B6, C2, C4, and C6 are the DF risk spatial distribution predicted by the C&S model. A2 is the DF risk spatial distribution predicted by the socioeconomic model. Please note that the socioeconomic model in the future is not considered here because the spread of mosquito vectors cannot be separated from climate change, and the simulation results of the socioeconomic model is the worst based on previous results. https://www.resdc.cn/data.aspx?DATAID=201.

The remaining 75% of the 2014 DF case data were compared with the prediction model results in the current scenario to calculate the number and proportion of cases in each risk area (Table 2). DF cases had a significantly stratified distribution in different risk areas, indicating that the model had a better effect on dividing the risk of the DF epidemic. Compared with the climatic or socioeconomic variable model, the area of the risk region identified by the C&S variable model was smaller (Fig 4A1, 4A2, 4A3 and Table 2), accounting for only 38.13% (less than the 51.01%/39.89% of the climatic/socioeconomic variable model). The ratio of the DF cases number covered in the high-risk region to the risk region area (i.e., density of DF cases) reached 15.09 cases per km^2^, which was much higher than 10.71/6.79 cases per km^2^ of the climatic/socioeconomic variable model. These results illustrated that the dengue Maxent model, which considers C&S variables, can be more effective in predicting the distribution of DF risk zones with more statistical significance when the risk region classification criteria are the same.

### Spatial patterns of DF risk

The spatial distribution of DF risk areas simulated in the different variable models had similar overall characteristics and obvious local characteristics differences (Fig 4). The potential high-risk areas of the current period were mainly located at the junction of Guangzhou and Foshan, north-central Zhongshan City, northeast of Jiangmen City, and the coastal borders of Shenzhen and Zhuhai. The low and moderate risk regions were scattered across the PRD. The scope of the DF risk areas in the future will expand and the high risk region will spread to the western of Dongguan and coastal areas of Zhuhai. Moreover, there is a significant difference in the spatial distribution of the epidemic risk between the climatic model and the C&S model. The risk areas of the climatic model are contiguously concentrated, while the internal differences in the risk areas of the C&S model are distinct and discrete. In other words, climatic variables mainly describe the overall pattern of the dengue risk area, while socioeconomic variables determine the details of the DF spatial distribution.

**Fig 5:**
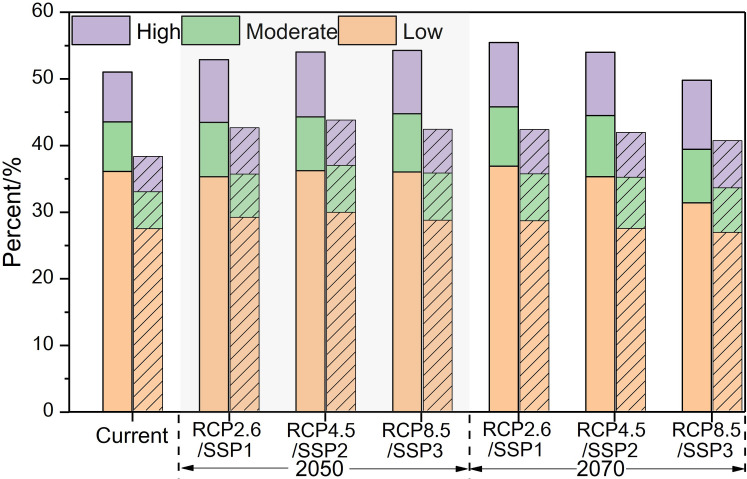
The DF risk area proportion of the climatic model and C&S model in the PRD. The left represents the climatic model. The right represents the C&S model.

In the different RCP/SSPs scenarios, the proportion of the risk region in the C&S models is lower than that of the climatic variable models. Under the RCP4.5/SSP2 scenario in 2050 (Figs 4 and 5 and S2 Table), the proportion of the moderate and high-risk zone will increase the most compared with other scenarios and the areas are mainly distributed in the junction of Guangzhou-Foshan, north-central Zhongshan City, and central-western Dongguan City. By 2070, the moderate and high risk zone will furthered expand to the Fenggang Town in Dongguan and the Longgang District in Shenzhen. However, under the RCP8.5/SSP3 scenario, the area of the risk zone is the lowest of the three scenarios. We predict that 45.13 million people (60.17% of the PRD’s population) in 2050 and 48.47 million people (63.78% of the PRD’s population) in 2070 under the RCP4.5/SSP2 will live in areas that are at the high and moderate risk for dengue transmission (S3 Table). These results indicate that the C&S model can effectively identify more detailed risk distribution characteristics within the region than the climatic model. In the 21^st^ century, DF risk varies under the different scenarios. Under the RCP4.5/SSP2 scenario, high and moderate risk of DF in the PRD region will continue to increase and 63.78% of the population of PRD in 2070 will live in these risk zones.

**Fig 6:**
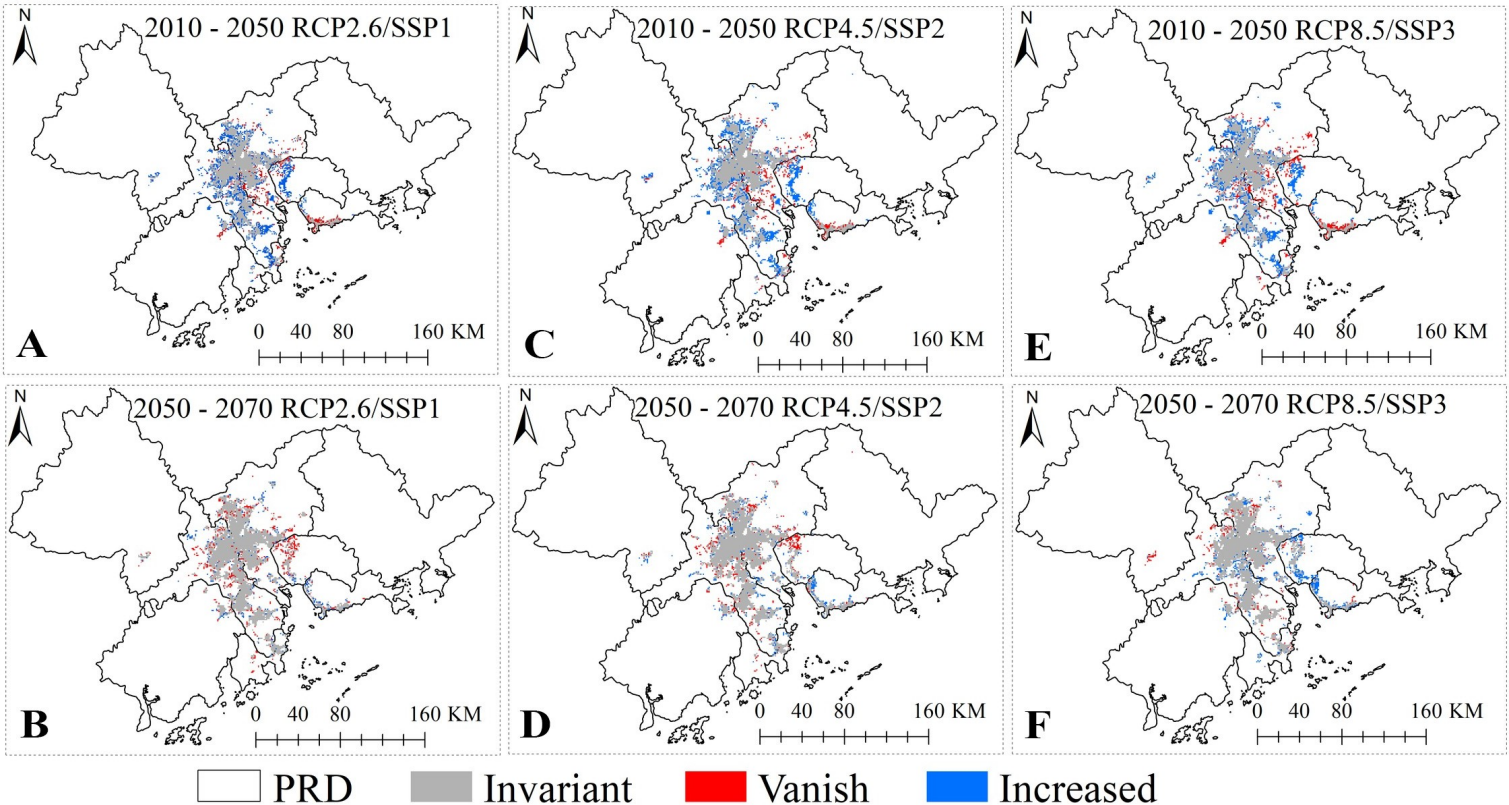
Spatial evolution of the DF high-risk areas under the different RCP/SSPs scenario in the C&S model. https://www.resdc.cn/data.aspx?DATAID=201.

According to the change trend of the DF high-risk areas, from the current time period to 2050 (Fig 6A, 6C and 6E), the reduced high-risk area will account for 9.54%–12.05% of the total change area. This change will be mainly distributed in the Panyu District of Guangzhou, the Xinhui District of Jiangmen, and the Nanshan District of Shenzhen. The newly expanded risk region accounts for 27.50%–29.87% of the total change areas, and are mainly distributed in the Huadu District of Guangzhou, the Nanhai and Shunde District of Foshan, the Shiqi District and Dong District of Zhongshan, and the urban areas of Houjie Town and Humen Town of Dongguan. From 2050 to 2070 (Fig 6B, 6D and 6F), the high-risk area will change little, and the new high-risk area will only account for 6.24% –12.86% of the total change region and will be mainly distributed in the Humen Town, Songgang Town of Baoan District, Shajing Town and Fuyong Town at the junction of Dongguan and Shenzhen. We predict that the population lived in the DF high-risk area, from current to 2070, will continue to increase, and reach a maximum of 30.78 million (40.5% of the PRD’s population) under the RCP 4.5/SSP2 scenario in 2070 (S3 Table).

## Discussion

Based on the Maxent model integrating DF cases data and climatic and socioeconomic environmental data in the PRD region, we explored and compared the distribution, spreading trend, and temporal-spatial pattern of DF risk in the different current and future scenarios. Several notable findings provide information for public health policymakers to develop long-term planning for the preventions and interventions of this disease.

The use of a fine spatial scale (1 km × 1 km; roughly equivalent to a small township or street) was a critical step towards the precise identification of the spatial patterns of DF risk in the PRD. Many studies of DF epidemic risk at large scales (regional, municipal, or county) found that climatic variables were relatively more important [46–48]. At a fine scale, we found that socioeconomic factors had the greatest impact on the spread of DF risk which was consistent with other fine-scale studies [49–52]. This discrepancy may be due to the fact that, at a fine spatial scale, socioeconomic factors have greater spatial heterogeneity than climatic variables, which are more effective for analyzing the spatial differentiation of DF on a small scale. With the development of the Guangdong-Hong Kong-Macao Greater Bay Area [21], the fine scale prediction results of DF risk spatial distribution in the PRD combined with the economic development model allow more conducive to accurate prevention and control information for public health policymakers, vaccine developers, and vector control specialists.

Under different RCP/SSPs scenarios in the future, socioeconomic factors have the greatest influence on the dengue epidemic in the PRD. This indicates that urban development has an important impact on the prevalence and transmission of DF and road density and that population density is a critical factor (Table A in S3 Text). The DF epidemic risk in the PRD increases as roads increase to 10 km/km^2^ and population density increases to approximately 3,500/km^2^ (Fig A in S3 Text). A developed road network improves transportation convenience, expands population mobility (including patients with dengue fever) and increases the probability of human contact with mosquitoes [11]. Moreover, the activity range of *Ae*. *albopictus* generally does not exceed 400 m, and its breeding and spawning are mainly concentrated in buildings or crowded areas [53,54]. This means that a high-density human population provides ideal conditions for DF transmission. There are numerous old urban areas and urban villages at the junction of Guangzhou and Foshan. These regions are commonly featured by poor sanitation, overcrowded population, absent infrastructure, and some environmental pollution due to the development is neither authorized nor planned, resulting in conditions highly suitable for the reproduction of *Ae*. *albopictus* [23]. Furthermore, construction land and GDP are also very important influencing factors, but it is worth mentioning that in the future scenario, the contribution of GDP will decline rapidly. This effect may be related to the continuous development of cities, the rapid economic development, and the improvement of living conditions and health status, leading to the antagonistic relationship between DF epidemics and GDP [55]. Accordingly, we cautiously recommend that, in addition to focusing on old urban areas for future epidemic prevention and control, urban regions with high density population and road networks within a certain range should also be considered.

Previous studies have estimated that with continued global warming, about 3–5 billion people would be at risk of dengue transmission by the end of 21^st^ century [3,56]. We predict that 48.47 million people will be exposed to high and moderate DF risk under the RCP4.5/SSP2 scenario in 2070, and this is 1.89 times the population exposed to DF risk in 2015 (S3 Table). This growth will be driven by population growth in existing endemic areas rather than the spread of DENV to new areas. In addition, the risk zone area is the smallest of the three scenarios under the RCP8.5/SSP3 scenario. This may be result from the limited range of temperatures suitable for the distribution of DF risk [11,57]. Accordingly, the future trajectory of DF in the PRD is highly dependent on which RCP/SSP scenario is realized. This suggests that curbs in emissions and more sustainable socioeconomic growth targets offer hope for limiting the future impact of dengue, and effective prevention and control need to continue to be strengthened at the junction of Guangzhou-Foshan, north-central Zhongshan city, and central-western Dongguan city.

Any long-term future projection is subject to a range of assumptions and limitations. In particular, we assume the stationarity of the effects and interactions of drivers of dengue transmission, the absence of innovations and improvements in dengue control, and the invariance of the imported DF cases. Due to the symptoms of DENV infection in human vary from inapparent or mild febrile illness to severe and fatal hemorrhagic disease, the reported DF case data used in this study may be underreported [58,59]. We did not include imported DF cases in our models, even though imported cases typically trigger DF epidemics in the PRD, and we did not consider the possible future spread of *Ae*. *aegypti* and *Ae*. *albopictus* which could influence DF distribution in the future [60]. Because we applied global data sets with inherent uncertainties to make relatively fine-scale predictions within a special region in China, errors may propagate with each added data set. The long-term prediction of populations and GDP in this study need to be further verified and comparison of multiple model predictions could be also added to further improve the fitting effect of the model. Despite these limitations, projections using these data have considerable public health value, and systematically derived projections provide an evidence base that can be updated through time for prioritizing resources and assisting in long-term planning.

## Supporting information

S1 Fig Number of DF cases reported in the PRD from 2003 to 2014. (TIF)

S2 Fig Total population and GDP in the Pearl River Delta from 2010 to 2100 under the five SSPs. (TIF)

S1 Text Spatial distribution simulation of socioeconomic development. (DOCX)

S2 Text Principal component analysis among different variables. (DOCX)

S3 Text Contribution of environmental variables in the C&S model. (DOCX)

S1 Table Area and proportion of different land use types from 2015 to 2070. (DOCX)

S2 Table The DF risk area proportion of the climatic model and C&S model in the PRD from current to future (%). (DOCX)

S3 Table Total population at the risk of DF in the PRD from current to future (ten thousands person). (DOCX)
