# Supplementary figures and images for: Increasingly expanded future risk of dengue fever in the Pearl River Delta, China
Source: PLoS Negl Trop Dis. 2021 Sep 24;15(9):e0009745. doi: 10.1371/journal.pntd.0009745 (PMC8462684; doi:10.1371/journal.pntd.0009745)

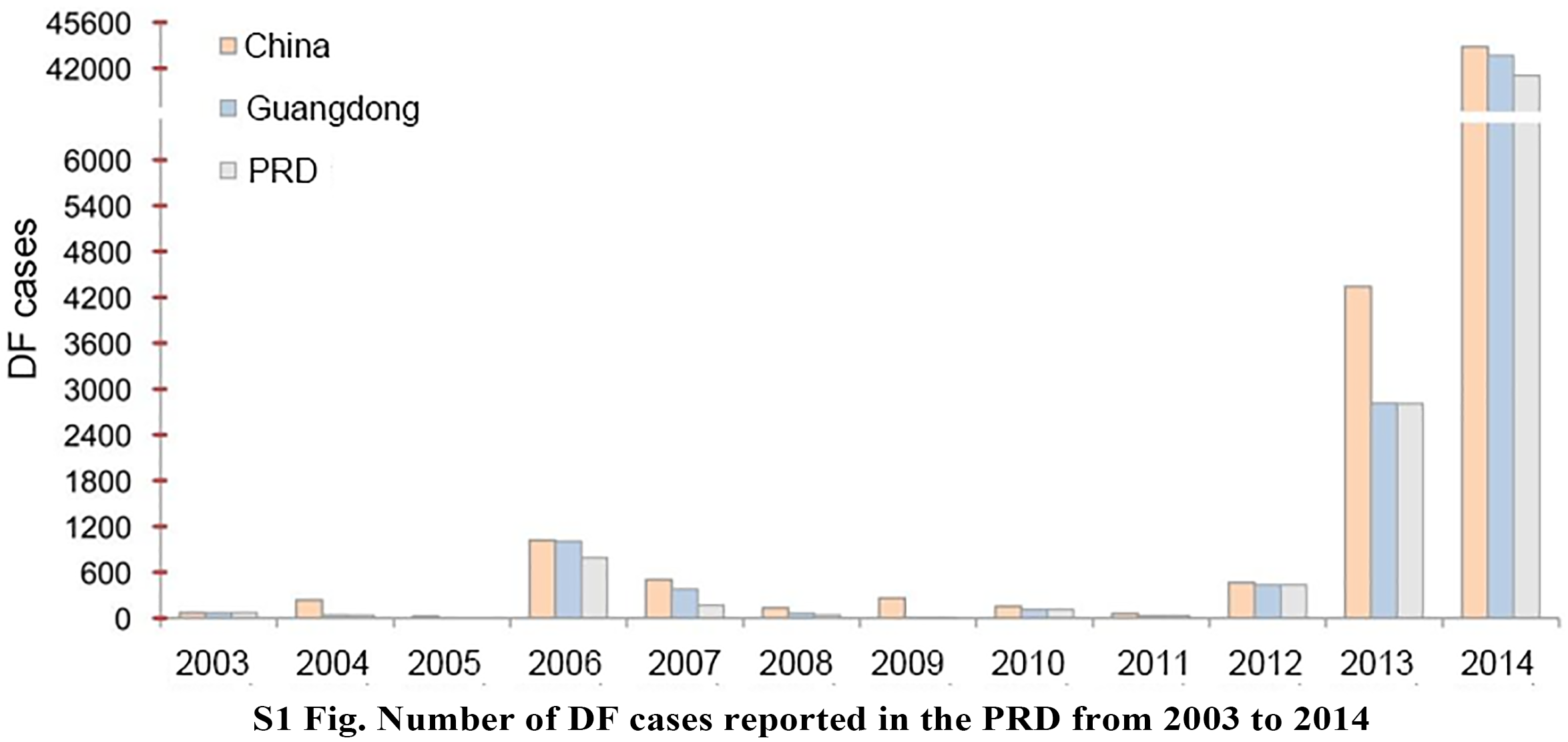

Supplement: S1 Fig — (TIF) [file pntd.0009745.s001.tif]

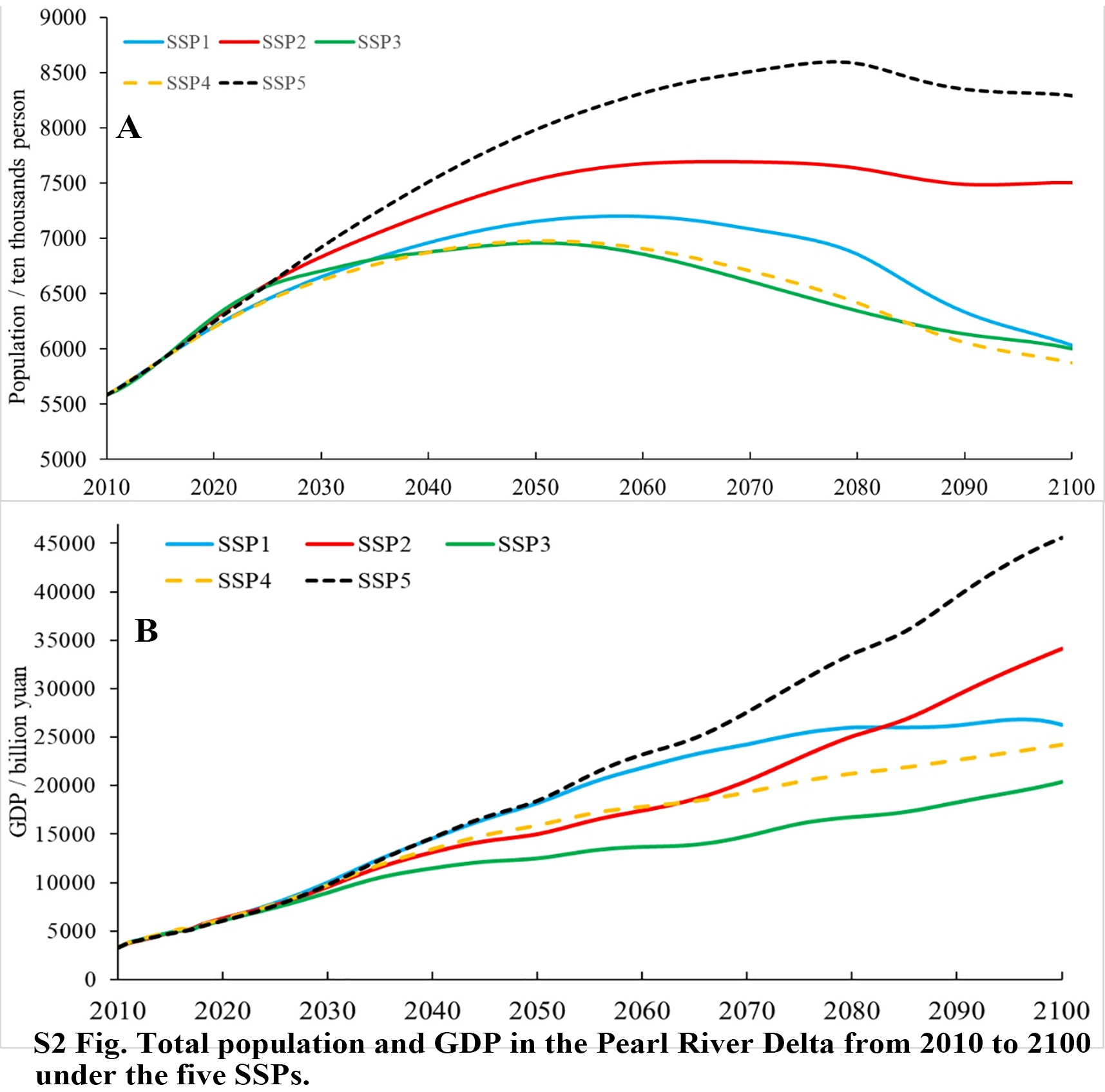

Supplement: S2 Fig — (TIF) [file pntd.0009745.s002.tif]
