# Supplementary material for: Increasingly expanded future risk of dengue fever in the Pearl River Delta, China
Source: PLoS Negl Trop Dis. 2021 Sep 24;15(9):e0009745. doi: 10.1371/journal.pntd.0009745 (PMC8462684; doi:10.1371/journal.pntd.0009745)
Supplement: S1 Text — (DOCX) [file pntd.0009745.s003.docx]

**Spatial distribution simulation of socioeconomic development**

① *Population Per Grid*

First of all, the dominant factors affecting the spatial distribution of population included climate, topography, hydrology, LULC, traffic conditions, etc. Among them, land use types (especially cultivated land, residential sites and urban industrial and mining land) had the greatest impact on population distribution [1,2]. It was affected by natural factors such as topography, hydrology and climatic conditions, and was also closely related to human social and economic activities. Compared with other factors, LULC can reflect the micro-population spatial distribution. As such, LULC was more effective than others when we spatialized population data.

Subsequently, we calculated the correlation between the different land use index (i.e., the ratio of different land use area to county area) at county level and the mean population density at county level (Table A). It is found that the population density in the PRD has the strongest correlation with urban land, followed by cultivated land, which is not related to the water and unused land. According to the order of correlation, we selected the county-level urban index, cultivated land index, grass index, forest index, wetland index and other index as independent variables, and county-level population density as the dependent variable. For example, the established model in 2050 was shown in Equation (1), R^2^ reach 0.988, Adjust R^2^ reach 0.986.

Pop=exp(11.012*R_1_+6.227*R_2_+10.564*R_3_+5.996*R_4_+6.226*R_6_+16.131*R_8_+9.106*R_9_)

(1)

where Pop denotes the population density, R_1_ is the urban land index; R_2_ is the cultivated land index; R_3_ is the Grass index; R_4_ is the forest index; R_6_ is the wetland index; R_8_ is the rural residential index; R_9_ is the other construction land index.

Finally, based on the established model (Equation 1), we calculated the population density per 1×1-km grid by linking the obtained future land use data and adjusted the total population for each scenario (SSPs 1-3) and year (2050, 2070) according to the results of S2a Fig. This method has been widely used in many studies [3-5].

**Table A. Relationship between population densities at the county level and land use ratios.**

| Lands use type | Urban  Land  index | CL Index | Grass Index | Forest Index | Water Index | Wetland Index | UL Index | R_R Index | Other Index |
| --- | --- | --- | --- | --- | --- | --- | --- | --- | --- |
| 2050 | 0.814** | -0.608** | -0.404** | -0.475** | 0.095 | -0.329* | -0.130 | -0.388** | -0.185 |
| 2070 | 0.815** | -0.623** | -0.405** | -0.486** | 0.122 | -0.328* | -0.129 | -0.374** | -0.195 |

② *GDP Per grid*

We next calculated GDP per capita by dividing GDP by the total population for each scenario (SSPs 1-3) and year (2050 and 2070). Then, we were able to multiply GDP per capita by population density and produce GDP per 1×1-km grid.

**Reference:**

1. Liao S-b, Li Z-h. Relationship between population distribution and land use and spatialization of population census data. Resources and Environment in the Yangtze Basin. 2004;13(6):557-61.

2. Tian Y, Chen S, Yue T, Zhu L, Wang Y, Fan Z, et al. Simulation of Chinese population density based on land use. ACTA GEOGRAPHICA SINICA-CHINESE EDITION-. 2004;59(2):283-92.

3. Liu H, Jiang D, Yang X, Luo C. Spatialization approach to 1 km grid GDP supported by remote sensing. Geo-information Science. 2005;2:026.

4. Xiao-gu S, Man-chun L, Yong-xue L, Wei L, Lu T, editors. A Semi-automation Road Extraction Approach Based on Fast Marching Method and Mean Shift Algorithm. 2009 WRI Global Congress on Intelligent Systems; 2009: IEEE.

5. Kocabas V, Dragicevic S. Bayesian networks and agent-based modeling approach for urban land-use and population density change: a BNAS model. Journal of geographical systems. 2013;15(4):403-26.
