# Supplementary material for: Increasingly expanded future risk of dengue fever in the Pearl River Delta, China
Source: PLoS Negl Trop Dis. 2021 Sep 24;15(9):e0009745. doi: 10.1371/journal.pntd.0009745 (PMC8462684; doi:10.1371/journal.pntd.0009745)
Supplement: S2 Text — (DOCX) [file pntd.0009745.s004.docx]

**Principal Components Analysis (PCA)** **among different variables**

First of all, according the correlations between all pairs of variables (Fig A), there was a high correlation among some variables, particularly climatic ones. Accordingly, principal components analysis (PCA) was then used to produce independent predictor variables from the climatic variables. Preliminary results (Table A) suggested that the first four principal components sufficiently represented 95.25% of the variance of all original continuous variables. The first principal component (C1) represented the majority of the climatic variables, and the rest of the principal components represented the remainder of the climatic variables. Therefore, one PCA models were generated. This model used the first four principal components (C1–C4) derived from the climatic variables only, as well as the original socioeconomic variables (Table A).


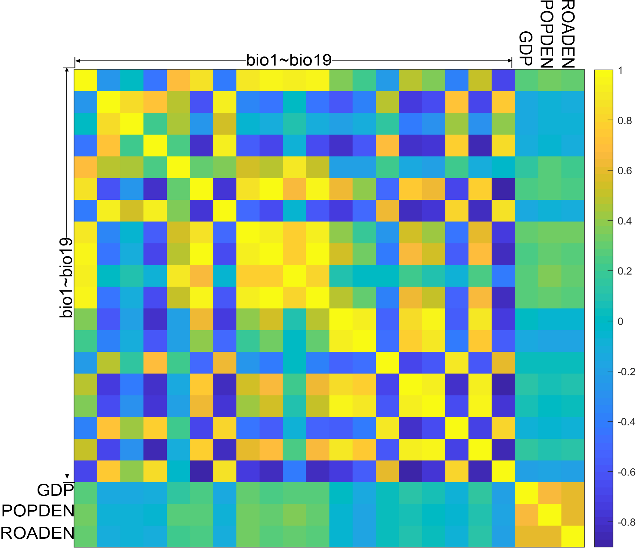


**Fig A. Matrix of correlations between all pairs of variables.**

**Table A. Standardized principal components analysis of climatic variables.**

|  | C1 | C2 | C3 | C4 |
| --- | --- | --- | --- | --- |
| EigenValue | 0.99 | 0.22 | 0.16 | 0.06 |
| Percent of EigenValues | 65.78 | 15.13 | 10.55 | 3.79 |
| Cumulative | 65.78 | 80.91 | 91.46 | 95.25 |
| Eigenvectors | | | | |
| Bio1 | 0.11 | 0.34 | 0.01 | 0.11 |
| Bio2 | -0.24 | 0.16 | 0.45 | -0.12 |
| Bio3 | -0.11 | 0.20 | 0.54 | -0.55 |
| Bio4 | -0.25 | 0.01 | 0.05 | 0.44 |
| Bio5 | -0.03 | 0.39 | 0.15 | 0.18 |
| Bio6 | 0.18 | 0.23 | -0.08 | -0.05 |
| Bio7 | -0.28 | 0.07 | 0.26 | 0.25 |
| Bio8 | 0.17 | 0.41 | -0.05 | 0.00 |
| Bio9 | 0.15 | 0.28 | -0.02 | -0.02 |
| Bio10 | 0.05 | 0.38 | 0.02 | 0.25 |
| Bio11 | 0.15 | 0.30 | -0.03 | 0.01 |
| Bio12 | 0.34 | -0.15 | 0.30 | 0.06 |
| Bio13 | 0.27 | -0.24 | 0.46 | 0.28 |
| Bio14 | -0.13 | 0.05 | 0.02 | 0.38 |
| Bio15 | 0.33 | -0.05 | 0.02 | 0.10 |
| Bio16 | 0.35 | -0.16 | 0.22 | 0.15 |
| Bio17 | -0.15 | 0.02 | 0.15 | 0.26 |
| Bio18 | 0.36 | -0.01 | 0.10 | -0.03 |
| Bio19 | -0.28 | -0.13 | 0.13 | 0.02 |

Among the all models (model A1-A3 and model B), model A3 had the highest AUC value and identified the smallest amount of the PRD as high risk (8.19%), and accounted for the largest percentage of DF cases. This evidence suggested that model A3 (i.e., all Climatic and Socioeconomic variable) is the best predictor of DF risk. Accordingly, it may be difficult to determine which variable can be deleted, our study used the all climatic and socioeconomic variables as the input variables of Maxent model.

**Table B. The value of AUC in the** **climatic principal components and socioeconomic model.**

| Model | Variables | Mean AUC Value | | DF cases in given risk area (% of Cases/ % area of the PRD) | | | |
| --- | --- | --- | --- | --- | --- | --- | --- |
|  |  | Training AUC | Test AUC | Zero | Low | Moderate | High |
| B | Climatic principal components (C1-C4) and Socioeconomic | 0.858 | 0.853 | 0.39/56.81 | 3.76/30.22 | 5.43/6.73 | 90.42/6.24 |
