# Supplementary material for: Increasingly expanded future risk of dengue fever in the Pearl River Delta, China
Source: PLoS Negl Trop Dis. 2021 Sep 24;15(9):e0009745. doi: 10.1371/journal.pntd.0009745 (PMC8462684; doi:10.1371/journal.pntd.0009745)
Supplement: S3 Text — (DOCX) [file pntd.0009745.s005.docx]

**Contribution of environmental variables in the C&S model.**

During the calculation of the model, each variable makes a different contribution to the model. In the current time period, the dominant influencing factors are road network density (GDP), GDP, and population density, with the contribution rate of three variables exceeding 83%, and the total contribution rate of climatic variables are only 13.6%, which indicates that socioeconomic variables have a greater impact on the distribution of epidemic risk, especially road density and GDP. Comparing the variable contribution rates of different future scenario models, socio-economic variables will still be the dominant factor, of which population density shows the largest contribution rate, followed by road network density, GDP, and land use types; and the contribution rate of climatic variables has increased by no more than 18.6 %. These results showed that in the current and future time periods, socio-economic variables will always be the dominant factor affecting the spatial distribution of DF epidemics, among which road network density and population density will always make a greater contribution.

**Table A.** **Percent contribution of environmental variables in the C&S model.**

| Variables | Percent contribution | | | | | | |
| --- | --- | --- | --- | --- | --- | --- | --- |
|  | A3 | B2 | B4 | B6 | C2 | C4 | C6 |
| bio1 | 0.4 | 0.5 | 0.1 | 0.3 | 0.3 | 0.3 | 0.1 |
| bio2 | 0.2 | 0.4 | 0.6 | 0.4 | 0.5 | 0.7 | 1.2 |
| bio3 | 1.0 | 0.2 | 0.1 | 0.6 | 0.3 | 0.3 | 0 |
| bio4 | 0.3 | 0.2 | 0.1 | 0.1 | 1.5 | 0.3 | 0.7 |
| bio5 | 0.3 | 0.9 | 2.0 | 0.6 | 0 | 2.0 | 0.4 |
| bio6 | 1.6 | 0.1 | 0.2 | 0.1 | 0.3 | 0.3 | 0.1 |
| bio7 | 0.7 | 0.4 | 0.8 | 0.8 | 0.2 | 0.5 | 0.2 |
| bio8 | 1.8 | 1.5 | 0.2 | 1.6 | 0.2 | 0.1 | 0.1 |
| bio9 | 0.1 | 0.4 | 0.6 | 0 | 0.6 | 0.7 | 0.1 |
| bio10 | 0.8 | 0.3 | 0.5 | 0.5 | 0.5 | 0.7 | 0.2 |
| bio11 | 0.4 | 0.4 | 0.4 | 0.2 | 0.3 | 1.5 | 0.7 |
| bio12 | 1.1 | 0.6 | 0.7 | 0.2 | 1.1 | 2.1 | 0.5 |
| bio13 | 2.2 | 1.2 | 0.6 | 0.6 | 0.4 | 0.4 | 1.0 |
| bio14 | 0.5 | 2.3 | 1.7 | 1.7 | 1.4 | 0.2 | 0.6 |
| bio15 | 0.5 | 0.1 | 0.3 | 0.7 | 0.8 | 1.1 | 0.1 |
| bio16 | 0.3 | 1.8 | 0.2 | 0.3 | 0.5 | 0.7 | 4.8 |
| bio17 | 1.0 | 0.3 | 0.1 | 1.4 | 0.7 | 0.8 | 3.1 |
| bio18 | 0.2 | 4.8 | 6.5 | 7.6 | 4.1 | 4.9 | 2.0 |
| bio19 | 0.3 | 0.6 | 0.9 | 0.4 | 1.2 | 1.0 | 0.2 |
| GDP | 37 | 5.9 | 6.5 | 7.0 | 8.8 | 8.9 | 8.1 |
| ROAD | 24.1 | 24.9 | 23.7 | 21.3 | 30.4 | 27.7 | 29.0 |
| LUCC | 2.7 | 9.7 | 11.5 | 11.3 | 10.8 | 13.6 | 10.8 |
| POP | 22.6 | 42.5 | 41.7 | 42.3 | 35.1 | 31.2 | 36.0 |

Furthermore, by selecting the environment variable with higher contribution under the current scenario, according to the corresponding response curve generated, the response characteristics between the dominant variables and the dengue epidemic could be further analyzed. As shown in Fig A(a), the relationship between the risk of dengue outbreak transmission and GDP was complicated, with the emergence of two peaks (GDP reached 10,000 yuan and 46,000 yuan), and then the risk had been reduced. Fig A(b, d) indicated that the characteristics of the response relationship between population density, road network density and transmission risk were similar. When the population density is from 0 to 3500 person / km^2^ and the road network density is from 0 to 10 km / km^2^, the risk of epidemic spread increases with the increase of population density and road network density, after which the transmission risk remains basically unchanged. Based on the relationship between different land use types and the risk of epidemic transmission, urban land has the highest transmission risk, followed by rural residential land, other construction land, cultivated land, wetland, grassland and forest land. Fig A(e) showed that the risk of epidemic transmission decreases with the increase of rainfall in the wettest month, and there is almost no transmission risk when the rainfall is excessive. Fig A(f) illustrated that the transmission risk of the epidemic peaks at an average temperature of 27.5 ° C in the wettest season, and then the transmission risk decreases, reaching a valley at 28℃, and finally stabilizes.


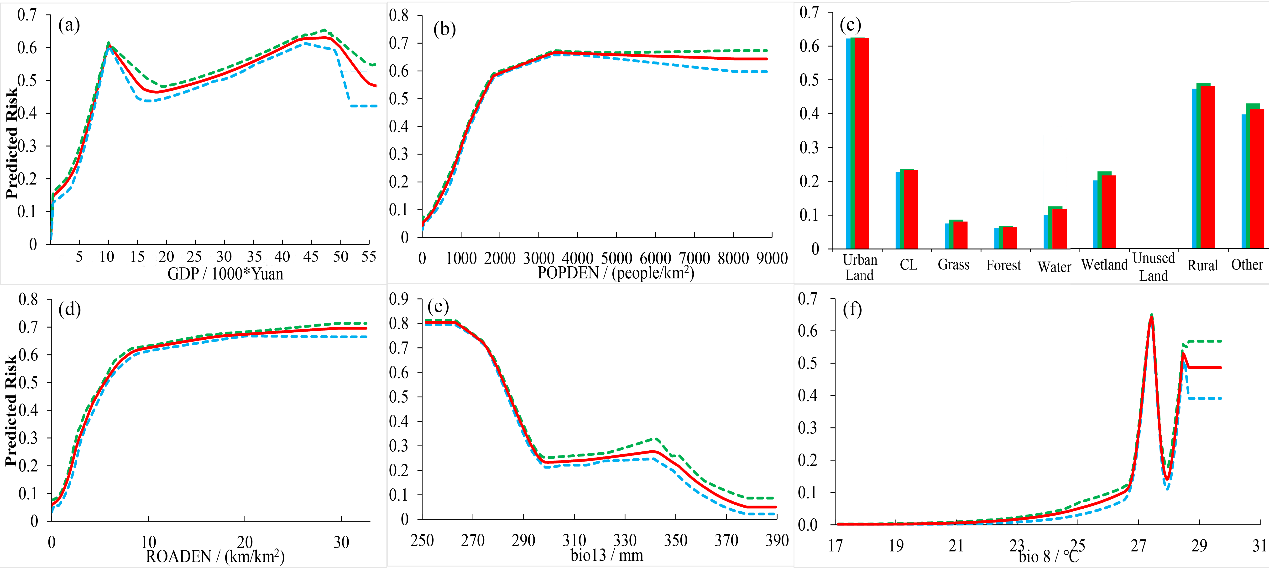


**Fig A. Response curves for the variables in the C&S model as related to the predicted risk of Dengue Fever outbreak.** Red line/bar indicated the mean values for the Maxent runs; Green dotted line/bar indicates the maximum values and blue dotted line/bar indicates the minimum value.
