# Supplementary material for: Increasingly expanded future risk of dengue fever in the Pearl River Delta, China
Source: PLoS Negl Trop Dis. 2021 Sep 24;15(9):e0009745. doi: 10.1371/journal.pntd.0009745 (PMC8462684; doi:10.1371/journal.pntd.0009745)
Supplement: S1 Table — (DOCX) [file pntd.0009745.s006.docx]

**S1 Table.** **Area and proportion of different land use types from 2015 to 2070.**

| LUCC | 2015 (km^2^) | Percent (%) | 2050 (km^2^) | Percent (%) | 2070(km^2^) | Percent  (%) |
| --- | --- | --- | --- | --- | --- | --- |
| Urban land | 4526.80 | 8.43 | 6647.16 | 12.37 | 6660.00 | 12.40 |
| Cultivated land | 12758.39 | 23.75 | 12147.96 | 22.61 | 12477.89 | 23.22 |
| Grass | 1138.00 | 2.12 | 1090.02 | 2.03 | 1070.84 | 1.99 |
| Forest | 27920.34 | 51.97 | 27352.74 | 50.91 | 27286.91 | 50.79 |
| Water | 1083.09 | 2.02 | 1040.26 | 1.94 | 1040.20 | 1.94 |
| Wetland | 2663.37 | 4.96 | 2249.57 | 4.19 | 2133.04 | 3.97 |
| Unused land | 7.06 | 0.01 | 6.97 | 0.01 | 6.80 | 0.01 |
| Rural residential | 1703.90 | 3.17 | 1441.38 | 2.68 | 1336.49 | 2.49 |
| Other | 1927.46 | 3.59 | 1753.29 | 3.26 | 1717.17 | 3.20 |
