# Supplementary material for: Increasingly expanded future risk of dengue fever in the Pearl River Delta, China
Source: PLoS Negl Trop Dis. 2021 Sep 24;15(9):e0009745. doi: 10.1371/journal.pntd.0009745 (PMC8462684; doi:10.1371/journal.pntd.0009745)
Supplement: S2 Table — (DOCX) [file pntd.0009745.s007.docx]

**S2 Table.** **The DF risk area proportion of the climatic model and C&S model in the PRD from current to future (%).** The left values represent the climatic model, while the right values represent the C&S model.

| Class | Current | RCP 2.6/SSP1 | | RCP 4.5/SSP2 | | RCP 8.5/SSP3 | |
| --- | --- | --- | --- | --- | --- | --- | --- |
|  |  | 2050 | 2070 | 2050 | 2070 | 2050 | 2070 |
| Low | 36.11/27.28 | 35.34/29.01 | 36.92/28.49 | 36.25/29.73 | 35.31/27.34 | 36.02/28.54 | 31.39/26.74 |
| Moderate | 7.46/5.53 | 8.13/6.45 | 8.87/7.04 | 8.06/7.05 | 9.20/7.65 | 8.76/7.09 | 8.05/6.67 |
| High | 7.44/5.32 | 9.43/6.98 | 9.66/6.62 | 9.74/6.82 | 9.48/6.73 | 9.51/6.56 | 10.38/7.09 |
| Total | 51.01/38.13 | 52.90/42.44 | 55.45/42.15 | 54.06/43.60 | 53.99/41.72 | 54.28/42.20 | 49.83/40.50 |

Note: RCP2.6/SSP1 represent global economic convergence and the introduction of resource-efficient technologies. RCP4.5/SSP2 represent low population growth and technological change. RCP8.5/SSP3 represent increasing population and regionally-oriented economic development.
