# Supplementary material for: Increasingly expanded future risk of dengue fever in the Pearl River Delta, China
Source: PLoS Negl Trop Dis. 2021 Sep 24;15(9):e0009745. doi: 10.1371/journal.pntd.0009745 (PMC8462684; doi:10.1371/journal.pntd.0009745)
Supplement: S3 Table — (DOCX) [file pntd.0009745.s008.docx]

**S3 Table.** **T****otal population at the risk of DF in the PRD from current to future (ten thousands person).**

| Class | Current | RCP 2.6/SSP1 | | RCP 4.5/SSP2 | | RCP 8.5/SSP3 | |
| --- | --- | --- | --- | --- | --- | --- | --- |
|  |  | 2050 | 2070 | 2050 | 2070 | 2050 | 2070 |
| Low | 1963 | 2328 | 2226 | 2373 | 2198 | 2202 | 2019 |
| Moderate | 881 | 1485 | 1549 | 1626 | 1769 | 1601 | 1276 |
| High | 1681 | 2715 | 2729 | 2887 | 3078 | 2544 | 2711 |
| Total | 4525 | 6528 | 6504 | 6886 | 7045 | 6347 | 6006 |
